# Diets for Dual Cardiovascular and Planetary Health: A Scoping Review

**DOI:** 10.1007/s11883-025-01344-5

**Published:** 2025-12-10

**Authors:** Sapna Peruvemba, Raquel Martinez, Joan Sabaté, Ujué Fresán

**Affiliations:** 1 School of Public Health, Loma Linda University, Loma Linda, CA USA; 2 Barcelona Institute for Global Health (ISGlobal), Barcelona, Spain; 3 Institute of Agrifood Research and Technology (IRTA), Caldes de Montbui, Spain

**Keywords:** Sustainable diet, Environmental impact, Cardiovascular disease, Eating pattern

## Abstract

**Purpose of Review:**

Most observational studies quantify the relationship between diet, cardiovascular disease (CVD), and environmental impacts independently, resulting in a fragmented understanding of sustainable diets. This review summarizes findings from observational studies assessing eating patterns and their *simultaneous* associations with environmental and CVD outcomes.

**Recent Findings:**

Plant-based diets, primarily those low in red meat, added sugars, and sodium, are associated with lower CVD risks. Environmental studies suggest that whole-food diets low in animal proteins typically have a lesser impact on greenhouse gas emissions (GHGe) and land use than diets high in animal proteins; however, they may increase water use.

**Summary:**

Predominantly plant-based diets were consistently associated with lower cardiovascular risk and reduced environmental impacts, though trade-offs were observed between healthiness and environmental sustainability, as well as across different environmental indicators. Further research is needed to determine how dietary patterns, cardiovascular health, and environmental outcomes align.

**Supplementary Information:**

The online version contains supplementary material available at 10.1007/s11883-025-01344-5.

## Introduction

Over the past 30 years, cardiovascular disease (CVD) has risen significantly and is now responsible for over one-third of deaths globally [1]. Diet plays a central role in modulating several key CVD risk factors—including blood lipids, blood pressure, and obesity—as well as influencing cardiovascular outcomes such as incidence of stroke, myocardial infarction, other cardiac events, and CVD-related mortality [2–5]. Diets rich in fruits, vegetables, whole grains, legumes, nuts and seeds, and fish, and low in red and processed meats, refined grains, and sugar-sweetened foods and beverages, have consistently been associated with a reduced risk of CVD [2]. Prominent examples of such dietary patterns include the Mediterranean diet, the DASH diet, and vegetarian diets, all of which have demonstrated protective effects against adverse cardiovascular outcomes [3–5].

Beyond human health, dietary choices have far-reaching environmental consequences. The food system—including production, distribution, consumption, and waste—is a major consumer of natural resources and a key contributor to environmental pollution [6]. The widespread adoption of environmentally sustainable diets is essential to keeping not only the food system within planetary boundaries [7], but also to achieving the targets set by the Paris Agreement [8]. As with health outcomes, the environmental impact of food varies. Animal-based foods generally have a greater environmental footprint compared to plant-based options, whether assessed per quantity, energy, or protein content [9–11]. Mathematical modeling and observational studies suggest that plant-based dietary patterns are typically more environmentally sustainable than meat-rich diets [12–15].

Previous reviews have mentioned that diets beneficial for CVD often align with those considered environmentally sustainable—particularly whole-food, plant-based dietary patterns [16–20]. However, these conclusions are largely based on studies that assessed health and environmental outcomes independently. Although healthy and environmentally sustainable diets share many principles, these two dimensions do not always align. Not all diets showing health benefits lead to a reduced environmental impact, and vice versa. For example, a recent trial found that greater adherence to the Mediterranean diet—despite its well-documented health benefits—was associated with increased environmental impacts for certain indicators [21]. In the same line, following the most environmentally sustainable diets may not guarantee health benefits [22]. Ultimately, the health and environmental impact of a diet depends on the specific foods consumed, and trade-offs between these outcomes have been reported. For instance, fish—valued for their cardioprotective long-chain omega-3 fatty acids [2]—can lead to significant environmental harm depending on the species targeted and fishing or aquaculture methods used [23, 24]. On the other hand, foods detrimental to cardiovascular health, such as sugar-sweetened beverages and ultra-processed snacks, may have lower environmental footprints than more nutrient-dense foods like fruits and vegetables [25]. Altogether, an integrated approach that assesses dietary patterns in relation to both CVD and environmental outcomes can deepen our understanding of truly environmentally sustainable and health-promoting diets.

The objective of this scoping review is to identify the existing literature on the simultaneous relationship between dietary patterns, environmental impacts, and CVD outcomes, with the aim of identifying dietary patterns that provide benefits for both cardiovascular health and environmental sustainability.

## Research Methods

### Study Design

We conducted a scoping review per the PRISMA-ScR (Preferred Reporting Items for Systematic Reviews and Meta-Analyses extension for Scoping Reviews) checklist to systematically identify published articles concerning dietary patterns, CVD outcomes, and environmental impacts within the same population [26]. The protocol has been previously published [27].

### Search Strategy

We conducted a comprehensive methodical search using two databases – PubMed and Web of Science – in February 2025 to identify relevant articles. The following search terms were tailored to each database, comprising the following: (“Diet” OR “dietary pattern”) AND (“environment” OR “environmental impact” OR “sustainability” OR “greenhouse gas emissions” OR “GHGe” OR “land use” OR “water use” OR “water consumption” OR “energy use” OR “footprint”) AND (“cardiovascular health” OR “cardiovascular mortality” OR “cardiovascular disease”). No filters were applied throughout the searching process to expand outcomes meeting the established criteria.

### Eligibility Criteria

The study selection was as follows: (i) study design: original research articles, including observational population-based studies and cohort studies; (ii) participants: adults (≥ 18 years); (iii): exposure: actual dietary patterns (e.g., vegetarian diet) or adherence to dietary indices (e.g., plant-based diet index); (iv) outcomes: cardiovascular (e.g., incidence of CVD events, CVD risk, CVD mortality) and environmental impact indicators (e.g., greenhouse gas emissions (GHGe), land use, water use, energy use), where CVD and environmental outcomes must be from the same study. We excluded the following: (i) randomized controlled trials or studies based on mathematical modeling, as they do not reflect real-world dietary patterns and (ii) studies focusing solely on CVD risk factors (e.g., high blood pressure) without reporting clinical outcomes, or reporting CVD outcomes alongside other health-related endpoints. We considered composite cardiovascular disease outcomes (i.e., CVD mortality, defined by ischemic heart disease, stroke, etc.) relevant to our investigation.

### Study Selection

After removing duplicates, two independent reviewers screened the titles and abstracts of search results to assess relevance. Full texts of potentially applicable articles were retrieved and assessed for eligibility by the same reviewers. Each reviewer independently summarized eligible studies in a spreadsheet. These summaries were then compared to finalize study selection. Discrepancies were resolved through discussion or, when necessary, consultation with a third reviewer.

### Data Extraction

Data from the selected studies was systematically extracted, comprising: author(s) and year of publication, study population (e.g., cohort name, sample size, age), region, environmental impact indicators (e.g., GHGe, land use, water use, energy use, eutrophication, fertilizer use), dietary assessment method (e.g., dietary records, recalls, food frequency questionnaires, adherence to dietary indices), CVD outcomes (e.g., incidence of CVD events, CVD risk, CVD mortality) apart from cerebrovascular diseases, and key findings.

## Results

### Literature Search

**Fig. 1:**
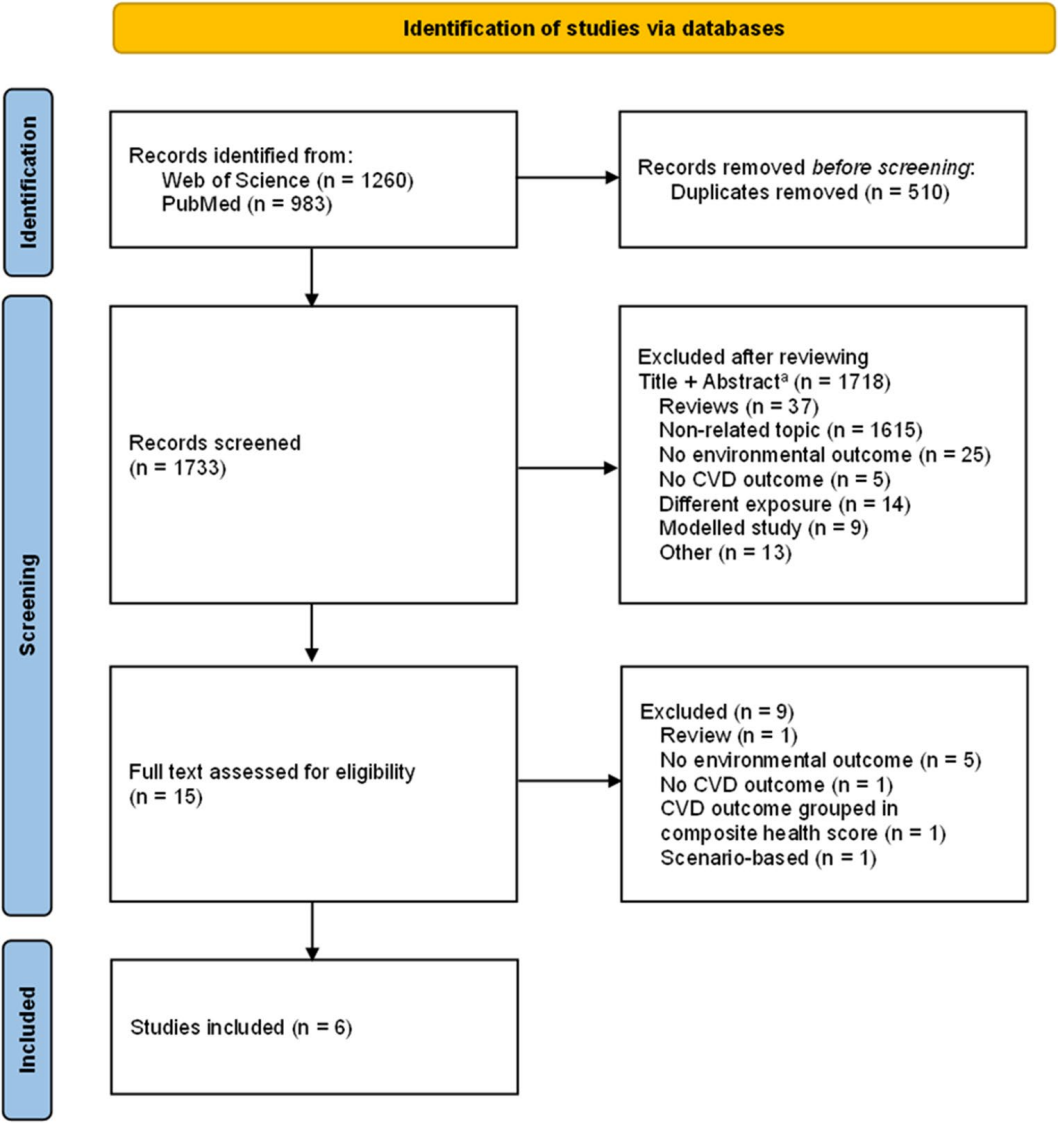
Flowchart of article selection process. Abbreviations: cardiovascular disease (CVD). ^a^ “Reviews” may include meta-analysis. Records were labeled as a “non-related topic” if they included only *one* component of interest (i.e., solely related to CVD, climate change, or diet). For the purpose of this review, CVD outcomes exclusive to stroke (cerebrovascular accidents) were deemed non-related. However, aggregated CVD outcomes—such as CVD risk defined by stroke, ischemic heart disease, and myocardial infarction incidence—were considered relevant. Articles examining the relationship between dietary patterns and environmental outcomes but not addressing CVD outcomes were categorized as “no CVD outcomes.” Conversely, studies investigating the link between diet and CVD outcomes without considering environmental outcomes were classified as having “no environmental outcomes.” Additionally, articles assessing the connections between food or nutrient-related exposures and either environmental or CVD outcomes were labeled “different exposure.” Notably, most records classified under “different exposure” referred to food-specific exposures (e.g., dairy intake) rather than dietary patterns. “Other” records include position papers, forum papers, qualitative studies, perspectives, and other article types

**Fig. 2:**
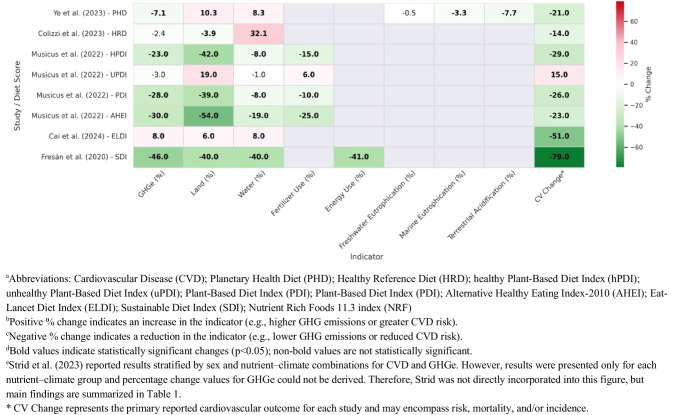
Changes in Environmental Indicators and Cardiovascular Risk Across Dietary Patterns^abcde^. ^a^Abbreviations: Cardiovascular Disease (CVD); Planetary Health Diet (PHD); Healthy Reference Diet (HRD); healthy Plant-Based Diet Index (hPDI); unhealthy Plant-Based Diet Index (uPDI); Plant-Based Diet Index (PDI); Plant-Based Diet Index (PDI); Alternative Healthy Eating Index-2010 (AHEI); Eat-Lancet Diet Index (ELDI); Sustainable Diet Index (SDI); Nutrient Rich Foods 11.3 index (NRF). ^b^Positive % change indicates an increase in the indicator (e.g., higher GHG emissions or greater CVD risk). ^c^Negative % change indicates a reduction in the indicator (e.g., lower GHG emissions or reduced CVD risk). ^d^Bold values indicate statistically significant changes (*p* < 0.05); non-bold values are not statistically significant. ^e^Strid et al. (2023) reported results stratified by sex and nutrient–climate combinations for CVD and GHGe. However, results were presented only for each nutrient–climate group and percentage change values for GHGe could not be derived. Therefore, Strid was not directly incorporated into this figure, but main findings are summarized in Table 1. * CV Change represents the primary reported cardiovascular outcome for each study and may encompass risk, mortality, and/or incidence

A total of 2,243 results were retrieved from PubMed and Web of Science combined (Fig. 1). After removing duplicates (*n* = 510), 1733 articles remained for eligibility screening. Ultimately, six articles met the inclusion criteria. The data extracted from these six studies are presented in Table 1, and a summary of main findings can be found in Fig. 2.

**Table 1:** Summary of the six cohort studies included in this scoping review

Author(s) (year)	Study Population (sample size)	Sample Demographics	Region	Dietary Data Collection	Dietary Assessment	Environmental Impact Indicators	Cardiovascular Disease Outcomes	Key Findings^a^
Ye et al. (2023)	Singapore Chinese Health Study (n = 63,257)	Singapore Chinese men and women aged 45 to 74	Singapore	FFQ	Planetary Health Diet (PHD) score	GHGe (kg CO₂ equivalent/day) Total water footprint (m³/day) Land use (m²/day)	CVD Mortality	Comparing highest to lowest quintile of: PHD scores: ↓ 21% CVD mortality HR (95% CI) 0.79 (0.73–0.85) P_trend_: <0.001 ↓ 7.1% GHGe Median [IQR] Q5: 2.6 [2.4-2.8] Q1: 2.8 [2.6-3.0] P_trend_: <0.0001 ↑ 8.3% total water footprint Median [IQR] Q5: 2.6 [2.4-2.7] Q1: 2.4 [2.3-2.6] P_trend_: <0.0001 ↑ 10.3% land use Median [IQR] Q5: 3.2 [2.9-3.7] Q1: 2.9 [2.6-3.3] P_trend_: <0.0001
Colizzi et al. (2023)	EPIC‐NL cohort (n = 35,496)	Dutch men and women, aged 20 to 70	Netherlands	FFQ	Healthy Reference Diet (HRD) score	GHGe (kg CO₂ equivalent/day) Land use (m^2^/year) Blue water use (m^3^/day) Freshwater eutrophication (kg phosphate equivalent/day) Marine eutrophication (kg nitrogen equivalent/day) Terrestrial acidification (kg sulfur dioxide equivalent/day	Total CVD events (fatal and nonfatal) Coronary heart disease (CHD)	Comparing highest to lowest quintile of: HRD scores: ↓ 14% CVD risk HR (95% CI) 0.86 (0.78–0.94) P_trend_: <0.001 ↓ 12% CHD risk HR (95% CI) 0.88 (0.78 - 1.00) P_trend_: 0.019 NS ↓ 2.4% GHGe (95% CI, −5.0 to 0.2) ↓ 3.9% land use (95% CI, −5.2 to −2.6) ↑ 32.1% blue water use (95% CI, 28.5 to 35.7) NS ↓ 0.5% freshwater eutrophication (95% CI, −2.6 to 1.6) ↓ 3.3% marine eutrophication (95% CI, −5.8 to −0.8) ↓ 7.7% terrestrial acidification (95% CI, −10.8 to −4.6)
Musicus et al. (2022)	Nurses’ Health Study II (n = 116,430)	US female nurses aged 25–42	United States	FFQ	Alternative Healthy Eating Index-2010 (AHEI) Plant-Based Diet Index (PDI) Unhealthy PDI Healthy PDI	GHGe, (kg CO₂ equivalent/day) Fertilizer needs (gNr/day) Cropland needs (m^2^/day) Irrigation water needs (m^3^/day	Incidence and Mortality: CVD CHD	Comparing highest to lowest quintile of: AHEI scores: ↓ 23% CVD risk RR (95% CI): 0.77 (0.66–0.89) P_trend_: <0.0001 ↓ 30% GHGe Adjusted Daily Means (95% CI)Q5: 2.6 (2.4 - 2.7) Q1: 3.7 (3.6 - 3.8) P_trend_: <0.0001 ↓ 25% fertilizer needs Adjusted Daily Means (95% CI) Q5: 73.4 (71.1 - 75.6) Q1: 97.8 (95.6 - 100.0) P_trend_: <0.0001 ↓ 54% cropland needs Adjusted Daily Means (95% CI) Q5: 16.6 (15.1 - 18.1) Q1: 35.9 (34.4 - 37.4) P_trend_: <0.0001 ↓ 19% irrigation water needs Adjusted Daily Means (95% CI) Q5: 0.62 (0.60 - 0.65) Q1: 0.77 (0.74 - 0.79) P_trend_: <0.0001 PDI scores: ↓ 26% CVD risk RR (95% CI): 0.74 (0.63–0.85) P_trend_: <0.0001 ↓ 28% GHGe Adjusted Daily Means (95% CI) Q5: 2.6 (2.5 - 2.8) Q1: 3.6 (3.5 - 3.7) P_trend_: <0.0001 ↓ 10% fertilizer needs Adjusted Daily Means (95% CI) Q5: 79.8 (77.4 - 82.2) Q1: 89.0 (86.6 - 91.4) P_trend_: <0.0001 ↓ 39% cropland needs Adjusted Daily Means (95% CI) Q5: 19.8 (18.2 - 21.4) Q1: 32.6 (31.0 - 34.2) P_trend_: <0.0001 ↓ 8% irrigation water needs Adjusted Daily Means (95% CI) Q5: 0.66 (0.63 - 0.69) Q1: 0.72 (0.70 - 0.75) P_trend_= 0.0014 Unhealthy PDIscores: ↑ 15% CVD risk RR (95% CI): 1.15 (1.00-1.33) P_trend_: 0.023 ↓ NS 3% GHGe Adjusted Daily Means (95% CI) Q5: 3.1 (3.0 - 3.2) Q1: 3.2 (3.1 - 3.3) P_trend_ = 0.94 ↑ 6% fertilizer needs Adjusted Daily Means (95% CI) Q5: 87.8 (85.4 - 90.2) Q1: 83.1 (80.7 - 85.5) P_trend_ = 0.0008 ↑ 19% cropland needs Adjusted Daily Means (95% CI) Q5: 28.8 (27.1 - 30.5) Q1: 24.3 (22.6 - 25.9) P_trend_ <0.0001 ↓ NS 1% irrigation water needs Adjusted Daily Means (95% CI) Q5: 0.68 (0.65 - 0.71) Q1: 0.69 (0.67 - 0.72) P_trend_ = 0.46 Healthy PDI scores: ↓ 29% CVD risk RR (95% CI): 0.71 (0.60–0.83) P_trend_ <0.0001 ↓ 23% GHGe Adjusted Daily Means (95% CI) Q5: 2.7 (2.6 - 2.8) Q1: 3.5 (3.4 - 3.6) P_trend_ <0.0001 ↓ 15% fertilizer needs Adjusted Daily Means (95% CI) Q5: 77.6 (75.3 - 80.0) Q1: 91.4 (89.0 - 93.7) P_trend_ <0.0001 ↓ 42% cropland needs Adjusted Daily Means (95% CI) Q5: 18.9 (17.3 - 20.5) Q1: 32.6 (31.0 - 34.2) P_trend_ <0.0001 ↓ 8% irrigation water needs Adjusted Daily Means (95% CI) Q5: 0.66 (0.63 - 0.68) Q1: 0.72 (0.69 - 0.75) P_trend_ <0.001
Cai et al. (2024)	China Health and Nutrition Survey cohort (n = 16,029)	Chinese men and women aged 18 to 65	China	Multiple (>1 day) 24-hour recalls	EAT-Lancet Diet Index (ELDI) score	GHGe (kg CO₂ equivalent/2000 kcal) Total water use (m^3^/2000 kcal), Land use (m^2^/2000 kcal)	Incidence of MI *or *stroke	Comparing highest to lowest quartile of ELDI score: ↓ 51% CVD Incidence HR (95% CI) 0·49 (0·37–0·66) P_linear trend_: <0·0001 ↑ 8% GHGe Mean (SD) Q4: 2.87 (1.3) Q1: 2.66 (1.21) P_trend_ <0.0001 ↑ 6% land use Mean (SD) Q4: 3.25 (1.51) Q1: 3.07 (1.33) P_trend_ = 0.0053 ↑ 8% total water use Mean (SD) Q4: 4.04 (1.54) Q1: 3.74 (1.44) P_trend_ <0.0001
Fresán et al. (2020)	Spanish SUN Cohort (n = 15,492)	Male and female Spanish university graduates, aged 20 and older.	Spain	Semi-quantitative FFQ	Sustainable Diet Index (SDI)^b^	Environmental impact index score^c^ Land use (m²/day) Energy use (MJ/day) Water consumption (liters/day) GHGe (kg CO_2_ equivalent/day)	Cardiovascular mortality	Comparing highest to lowest quartile of SDI score: ↓ 79% cardiovascular mortality HR (95% CI) 0.21 (0.05 - 0.85) P_trend_: <0.001 ↓ 40% land use Mean (SD) Q4: 5.34 (1.13) Q1: 8.96 (1.56) P_trend_: <0.001 ↓ 41% energy use Mean (SD) Q4: 13.1 (2.5) Q1: 22.1 (3.6) P_trend_: <0.001 ↓ 40% water consumption Mean (SD) Q4: 2810 (556) Q1: 4659 (762) P_trend_: <0.001 ↓ 46% GHGe Mean (SD) Q4: 2.49 (0.56) Q1: 4.59 (0.91) P_trend_: <0.001
Strid et al. (2023)	Northern Sweden Health and Disease Study participants (n = 80,335)	Swedish adults, aged 35 to 65 at baseline	Sweden	Semi-quantitative FFQ	Nutrient density with dietary climate impact^d^	GHGe^e^ (kg CO_2_ equivalents) Women: per 2000 kcal/day Men: 2500 kcal/day	MI	MI^f^ LNutr/HClim (reference) LNutr/LClim (HR, 95% CI) Women: NS ↑ 1.13 (0.94, 1.36) P = 0.180 Men: ↑ 1.19 (1.06, 1.33) P = 0.004 HNutr/LClim Women: NS ↑ 1.03 (0.85, 1.24) P = 0.762 Men: NS ↑ 1.05 (0.93, 1.17) P = 0.460 HNutr/HClim Women: NS ↑ 1.03 (0.84, 1.26) P = 0.780 Men: NS ↓ 0.99 (0.87, 1.12) P = 0.814 Climate Impact (GHGe) LNutr/LClim Men: 3.2 [2.9–3.5] Women: 2.6 [2.4–2.8] LNutr/HClim Men: 4.5 [4.1–5.2] Women: 3.6 [3.2–4.1] HNutr/ LClim Men: 3.2 [2.8–3.5] Women: 2.6 [2.3–2.8] HNutr/HClim Men: 4.4 [4.0–4.9] Women: 3.5 [3.2–3.9]

Alternative Healthy Eating Index-2010 (AHEI); Cardiovascular Disease (CVD); Coronary heart disease (CHD); EAT-Lancet Diet Index (ELDI) score; European Prospective Investigation into Cancer and Nutrition-Netherlands (EPIC‐NL) cohort; Food frequency questionnaire (FFQ); Greenhouse gas emissions (GHGe); Hazard Ratio (HR); Healthy Plant-Based Diet Index (hPDI); Healthy Reference Diet (HRD) score; High nutrient density with high climate impact (HNutr/HClim) diet; High nutrient density with low climate impact (HNutr/LClim) diet; Low nutrient density with high climate impact (LNutr/HClim) diet; Low nutrient density with low climate impact (LNutr/LClim) diet; Interquartile Range (IQR); Myocardial Infarction (MI); Nonsignificant (NS); Planetary Health Diet score (PHD score); Plant-Based Diet Index (PDI); Relative Risk (RR); Spanish Seguimiento Universidad de Navarra (SUN) Cohort; Standard Deviation (SD); Sustainable Diet Index (SDI); Unhealthy Plant-Based Diet Index (uPDI)

^a^The key findings reflect results from adjusted models per study

^b^This comprehensive dietary index score comprises three sub-indices, each addressing a specific dietary dimension. The nutritional quality sub-index is based on the 2015-2020 Dietary Guideline for Americans Index. The environmental impact index evaluates the ecological footprint of a diet by considering four factors: land use, water consumption, energy consumption, and GHGe. Market prices encompass the annual costs per FFQ food item, as per the Ministry of Industry, Tourism and Commerce of Spain. The total Sustainable Diet Index (SDI) score Ranges from 0 to 9, with each sub-index contributing between 0 and 3 points, where 0 represents the least favorable profile

^c^The environmental impact index score accounts for four ecological impacts: GHGe, land use, water consumption, and energy consumption. The score is reported as a mean and standard deviation per quartile of the Sustainable Diet Index, with a higher value representing greater resource use and environmental impact. Data concerning land use, energy use, water consumption, and GHGe was provided by the main author via personal communication

^d^Diets were evaluated and categorized according to nutrient density and climate impact, resulting in four eating patterns or dietary groups: low nutrient density with low climate impact (LNutr/LClim), low nutrient density with high climate impact (LNutr/HClim), high nutrient density with low climate impact (HNutr/LClim), high nutrient density with high climate impact (HNutr/HClim), where LNutr/HClim served as the reference. The diet quality was measured using the nutrient-density index — NRF11.3 — reflecting the Nordic Nutrition Recommendations and founded on the Nutrient Rich Foods index. The dietary climate impact component reflects GHGe. The authors established sex-specific cut-off values for NRF11.3 scores and climate impact to classify participants' eating patterns

^e^Values reported as median [IQR] and available in the article’s supplementary information (Supplementary Table 1), organized by gender

^f^MI HR data per eating pattern was provided by the authors via personal communication

### Study Characteristics

A total of six studies were included [28–33]. As shown in Table 1, the selected studies were all relatively recent, published between 2020 and 2024. Each was a population-based cohort study, with some focusing on specific occupational or educational groups, such as nurses [30] and university graduates [32]. Sample sizes ranged from 16,029 to 116,430 participants, with follow-up periods varying across studies; the median duration ranged from 9.86 to 23.4 years. Cohorts included adults aged 18 years and older from diverse regions (Sweden, Spain, China, the United States, the Netherlands, and Singapore). Most analyses included both males and females, though one study included only females [30].

**Fig. 3:**
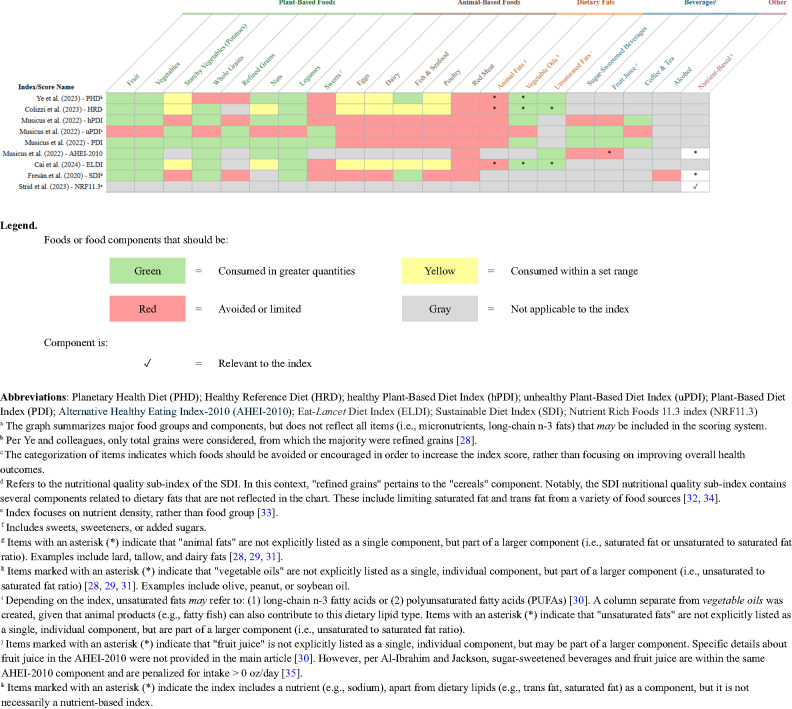
Outline of Food or Food Component Categorization per Index or Scoring Method^a^

Dietary intake was primarily assessed using validated food frequency questionnaires, though one study employed multiple non-consecutive face-to-face 24-hour dietary recalls [31]. All studies quantified dietary patterns through index-based scoring methods. Three studies focused on the EAT-Lancet recommendations but applied different dietary indices: the Planetary Health Diet (PHD) Score, the Healthy Reference Diet (HRD) Score, and the EAT-Lancet Diet Index (ELDI). Other indices used across the studies included, the Alternate Healthy Eating Index-2010 (AHEI-2010), and three versions of the Plant-based Diet Index—general (PDI), healthy (hPDI), and unhealthy (uPDI)—each targeted by a single study. Additional studies employed indices that integrated nutritional quality with other sustainability dimensions, such as environmental impact [32, 33] and market price [32]. Shared food groups and their classifications within the indices are shown in Fig. 3. Further details of each index are detailed in Supplemental Table 1.

Cardiovascular disease outcomes were assessed using a range of methods across studies, including clinical diagnoses (*n* = 2), hospital discharge records (*n* = 2), national health registries (*n* = 3), and mortality databases (*n* = 3). Most studies identified events or deaths according to standardized diagnostic codes from the International Classification of Diseases (ICD), with several using ICD-9 or ICD-10 codes to classify myocardial infarction (MI), coronary heart disease (CHD), and broader CVD categories (*n* = 4). Outcomes captured included both fatal and nonfatal cardiovascular events (*n* = 3), with some studies distinguishing between primary and secondary causes of death when evaluating mortality (*n* = 1).

Environmental indicators varied across studies, including GHGe (*n* = 6), land use (*n* = 4), cropland needs (*n* = 1), fertilizer needs (*n* = 1), freshwater and marine eutrophication (*n* = 1), terrestrial acidification (*n* = 1), and energy use (*n* = 1). Five studies included water-related indicators, but each of them used a different indicator (i.e., total water use, blue water use, total water footprint, water consumption, irrigation water needs). All studies estimated environmental impacts of reported dietary intake using life cycle assessment (LCA) methodology, although the scope of analysis varied. Some studies assessed environmental impacts only through the production and processing stages, excluding downstream activities like transportation, packaging, and household preparation [28, 30, 32]. Others adopted broader system boundaries, incorporating post-farm impacts such as packaging, transportation, retail storage, home preparation, and food loss along the supply chain [29, 31]. Studies also differed in their inclusion of food waste and consumer-level emissions: while some excluded these later-stage impacts [28, 30], others captured the full cradle-to-plate footprint [29]. As a result, the comprehensiveness of environmental impact estimates differed across studies.

All included studies used multivariable models, although the specific covariates varied across studies. Most commonly adjusted variables included age (*n* = 6), sex (*n* = 6), energy intake (*n* = 6), BMI (*n* = 6), smoking status (*n* = 6), physical activity (*n* = 6), and alcohol consumption (*n* = 4). Additional sociodemographic factors such as education level (*n* = 4), income (*n* = 1), and residential location (*n* = 1) were included in some studies. Several studies also adjusted for dietary or health-related behaviors, including dietary knowledge (*n* = 1), multivitamin or aspirin use (*n* = 1), oral contraceptive or menopausal status (*n* = 1), sleep duration (*n* = 1), and hours of TV viewing (*n* = 1). Clinical factors such as hypertension (*n* = 3), hypercholesterolemia (*n* = 1), diabetes (*n* = 1), and family history of MI (*n* = 2) were also included as covariates in some studies.

### Combined Health and Environmental Impacts of Dietary Patterns

As shown in Table 1; Fig. 2, our findings suggest that the highest (vs. the lowest) scores for AHEI-10, PDI, hPDI and SDI were associated with both lower risks of cardiovascular outcomes and reduced environmental impacts. Higher adherence to these diets was consistently linked to 23–46% reductions in GHGe [30, 32], 39–54% reductions in land use [30, 32], 10–25% reductions in fertilizer use [30] and 8–40% reductions in water-related indicators [30, 32]. These dietary patterns were also associated with 23–79% lower risks of CVD outcomes compared to those with lower adherence. On the contrary, the adherence to uPDI increased both the CVD risk (by 15%) and the environmental impacts, mainly fertilizer (by 6%) and cropland used (by 19%), although no statistical changes were observed for GHGe and irrigation water [30].

Beyond this alignment, some trade-offs were also noted—not only between health and environmental outcomes, but also among different environmental indicators (Table 1; Fig. 2). Specifically, the three studies assessing adherence to the EAT-Lancet diet reported reductions in health risk ranging from 14 to 51%. Nevertheless, the findings regarding environmental impacts differed substantially. Colizzi et al. reported a decrease in land use, marine eutrophication and terrestrial acidification, no significant change in GHGe and freshwater eutrophication, and an increase in water use [29]. In contrast, Ye et al. observed increases in both water and land use, despite reductions in GHGe [28]. Finally, Cai et al. found that GHGe, land use, and water-related impacts all increased [31]. On the other hand, Strid et al. (2023) found that among nutrient-poor diets in men, those associated with lower climate impacts were linked to a 19% greater risk of myocardial infarction. However, this association was not statistically significant in women, and no significant relationship was observed between myocardial infarction risk and either nutrient-rich or high-climate-impact diets [33].

## Discussion

This scoping review identified six studies that simultaneously assessed dietary patterns, environmental impacts, and cardiovascular disease outcomes within the same population. The current body of evidence remains insufficient to establish the most effective dietary pattern for optimizing both cardiovascular health and environmental sustainability, as each dietary pattern was evaluated in only one study—except for the EAT-Lancet recommendations, which were examined in three studies. Nevertheless, the findings suggest a consistent association between greater adherence to whole, plant-based diets and beneficial outcomes in both domains. Specifically, these dietary patterns were linked to a reduced risk of cardiovascular disease, as well as lower greenhouse gas emissions and land use.

Besides the limited number of studies that simultaneously address diet, cardiovascular disease, and environmental impact, several key findings warrant further attention.

### Variability in Benefits across Plant-Based Diets

The association between plant-based diets and both cardiovascular and environmental benefits was anticipated, as previous studies have reported similar findings—although many relied on mathematical modeling [36–44] or examined health and environmental outcomes separately [45–48]. However, the present review highlights the critical role that the specific types of plant-based foods consumed play in shaping these outcomes. For instance, while healthy plant-based diets were linked to both health and environmental benefits, adherence to unhealthy plant-based patterns resulted in increased environmental impacts (i.e., cropland and fertilizer use) and a higher risk of cardiovascular disease [30]. This can be explained by the composition of unhealthy plant-based diets, which typically include higher intakes of ultra-processed foods. Previous research also found that diets with greater land use were more likely to contain such products [22]. The inclusion of chocolate and high quantities of plant-based oils may partially account for the increased land requirements observed [9]. Furthermore, many of these ultra-processed foods are derived from commodity crops—such as corn or wheat—that are often grown in monocultures requiring intensive fertilizer use [49]. Additionally, their production typically involves high energy demands for processing and an increased reliance on packaging materials [50]. Consequently, diets rich in such products may impose a greater environmental burden compared to healthier plant-based diets. However, it is important to note that the level of processing alone does not fully determine the environmental sustainability of products, as the ingredient composition plays a more significant role [51]. While highly processed plant-based foods tend to have higher environmental impacts than less processed counterparts, they still generally have a lower footprint compared to animal-based products [51]. Ultimately, the environmental impact of diets is not solely determined by the consumption of ultra-processed foods, but mainly by the presence of animal-based foods and the dietary energy consumed [50]. On the other hand, from a health perspective, these diets are typically characterized by low nutritional quality—marked by high levels of added sugars, refined grains, and sodium, and low levels of dietary fiber and micronutrient-rich whole grains, fruits, vegetables, nuts, and seeds. These dietary characteristics are well-established risk factors for cardiovascular disease [52]. Previous studies have also highlighted the adverse health effects of unhealthy plant-based dietary patterns, linking them not only to increased cardiovascular risk [53], but also to many other outcomes, such as diabetes [54] and cancer [53, 55, 56], to name a few.

### Assessing Diet-Related Environmental Impact Requires Multiple Indicators

According to ISO standards guiding LCA methodology, all environmental impact indicators included in a selected characterization method should be reported to ensure transparency [57]. Our findings reinforce the importance of evaluating multiple environmental indicators, as trade-offs may arise between them. This was notably illustrated in Ye et al. (2023), who found that higher adherence to the Planetary Health Diet was associated with reduced GHGe, yet concurrently linked to increased total water footprint and land use [28]. Similarly, adherence to the Healthy Reference Diet was significantly associated with reductions in land use, marine eutrophication and terrestrial acidification; no associations with GHGe and freshwater eutrophication; and a blue water use increment [29]. As illustrated, reporting just one indicator may create misleading conclusions.

As highlighted in previous studies [58], we observed that GHGe, land use, and water-related indicators were the most frequently reported environmental outcomes, whereas others—such as energy use, fertilizer application, and eutrophication potential—received comparatively less attention. This imbalance may be partly attributed to the widespread reliance on secondary environmental impact data. Currently, most freely accessible environmental databases predominantly include those three indicators, limiting the scope of dietary environmental assessments. The lack of comprehensive and publicly accessible databases that encompass a broader range of environmental indicators—and account for the full life cycle of food products—represents a significant barrier to conducting holistic and nuanced assessments of dietary sustainability.

### Health and Environmental Trade-offs, and the Impact of Dietary Index Selection

Trade-offs between healthiness and environmental sustainability have been described in the literature [59], and our findings provide further examples of these divergences. The three studies assessing adherence to the EAT-Lancet diet consistently reported cardiovascular benefits. Nevertheless, the environmental outcomes were not aligned: while Colizzi et al. observed mainly environmental benefits—except for an increase in blue water use [29]—Ye et al. and Cai et al. reported higher environmental impacts among individuals with greater adherence to the EAT-Lancet diet [28, 31]. Indeed, the main interpretation of Strid et al. may be that dietary carbon footprint is not associated with health outcomes [33], a finding that has also been previously reported in the literature [22]. These findings have direct practical implications for health practitioners: promoting healthy diets does not necessarily guarantee a lower environmental impact. Health professionals require training to design and recommend dietary patterns that are both health-promoting and environmentally sustainable [60, 61].

Even though trade-offs between health and the environment are widely acknowledged, the increased environmental impacts associated with adherence to the EAT-Lancet diet in two of the three studies included in this scoping review are particularly surprising, given the consistent evidence from previous research suggesting that this dietary pattern is linked to lower environmental pressures [62–64]. The discrepancies may be partially explained by differences in the dietary composition among individuals in those specific cohorts (i.e., one Singapore Chinese, one Chinese, and the other Dutch) with higher versus lower adherence to the EAT-Lancet diet. However, another likely explanation is the use of different indices to measure adherence to the EAT-Lancet recommendations. A growing number of indices have been developed in recent years for this purpose, and a recent comparative evaluation identified those with better performance in capturing both health and environmental dimensions [65]. Notably, the index used by Colizzi et al. was among those recommended, whereas the indices applied by Ye et al. and Cai et al. were not highlighted as one of the most suitable tools [28, 29, 31]. The influence of dietary index selection on study outcomes is not unique to the EAT-Lancet diet; previous research has similarly demonstrated that different indices used to assess adherence to the Mediterranean diet can yield varying associations with health outcomes [66].

### Considerations for Adopting Sustainable Eating Patterns

To achieve the adoption of healthy diets with low environmental impact—especially in high-income countries—major changes to current dietary patterns are necessary, primarily by replacing animal-sourced and unhealthy foods with whole, plant-based options [62, 67]. However, identifying the traits of a sustainable dietary pattern is only the first step; the greater challenge lies in overcoming barriers to widespread adoption, including sociocultural, economic, and political factors [68].

Reducing meat consumption—a cornerstone of healthy, low-impact diets—remains one of the most resistant behaviors to change due to its deep ties to cultural traditions, religious practices, and social identity [69, 70]. Social norms, taste preferences, and entrenched habits further reinforce these patterns, making change both a behavioral and structural challenge [71, 72]. Affordability is another critical determinant of dietary choices [73]. While the EAT-Lancet diet’s estimated median cost of $2.84/day may be feasible in high-income countries, it remains unaffordable for many in low- and middle-income nations [74]. Even in industrialized nations, higher prices for foods perceived as sustainable, coupled with the ubiquity of low-cost ultra-processed foods, hinder adoption [75–77]. Misconceptions about the cost of plant-based diets and reluctance to pay more for such options further impede acceptance [78–80].

These challenges highlight the need to combine educational strategies with behavioral nudges and policy interventions to make sustainable, healthy diets both the most accessible choice and the new social norm [81]. However, competing interests among governments, industry, NGOs, and citizens often obstruct systemic change. Economic priorities, political inertia, and corporate influence frequently limit the policy, production, and trade shifts required to advance health-promoting, environmentally sustainable diets [82]. Political action is therefore pivotal—not only from food production and distribution perspectives, but also from the consumer side. Examples include improving access to sustainable foods, taxing unsustainable or unhealthy products and channeling the revenues into economic incentives or subsidies for whole, plant-based products, and embedding sustainability principles into dietary guidelines and public procurement, among others [72]. For these measures to be effective, whether educational, behavioral, or political, strategies must be locally adapted to align health and environmental sustainability with cultural acceptability [70].

### Strengths, Limitations and Perspective

This study has three major strengths. First, it focuses on self-selected dietary patterns, as opposed to relying solely on mathematical modeling, which may not fully reflect real-world consumption behaviors. Second, it specifically includes studies that assessed health and environmental outcomes simultaneously, allowing for a more integrated evaluation. This is important because results derived from separate studies may be influenced by cohort-specific dietary behaviors or the choice of dietary indices, as discussed earlier. Third, we included only studies that focused on cardiovascular disease as a distinct outcome, excluding those that used composite health endpoints [83]. This approach reduces the risk of diluting or misrepresenting the association between dietary patterns and cardiovascular outcomes by conflating them with other health conditions. Nevertheless, this scoping review is not without limitations. The most significant is the limited number of eligible studies, which, combined with considerable methodological heterogeneity—such as variation in the measurement of cardiovascular outcomes and differences in the system boundaries applied in environmental assessments—limits the ability to draw firm conclusions about the optimal dietary pattern for both cardiovascular health and environmental sustainability. However, as a scoping review, the main purpose is to summarize current evidence and highlight areas where further research is needed, rather than to draw firm conclusions. In doing so, this review underscores the urgent need for more integrated studies and highlights key methodological considerations that future research should address to advance the field. Specifically, future studies should apply validated and context-appropriate dietary indices (ideally, more than one), expand the assessment of dietary patterns with established potential for dual benefits—such as vegetarian and Mediterranean diets [84]—and rely on robust and standardized sources for both health and environmental data. In addition, assessments should incorporate multiple environmental indicators and consider the full life cycle of food products to capture the complexity of diet-environment-health interrelationships.

## Conclusions

The existing evidence indicates that whole plant-based diets may contribute to both reduced environmental impact and lower cardiovascular disease risk. However, this review reveals a significant gap in the literature: very few studies have simultaneously examined dietary patterns, environmental outcomes, and cardiovascular health within the same populations. To better understand the multifaceted impact of diet, future research should aim to assess a broader range of dietary patterns, cardiovascular risk factors, as well as environmental indicators. Such comprehensive approaches are essential to inform dietary recommendations that are beneficial for both human and planetary health.

## Key References

- Clark MA, Springmann M, Hill J, Tilman D. Multiple health and environmental impacts of foods. Proc Natl Acad Sci U S A. 2019;116(46):23357-62. 10.1073/pnas.1906908116.
  - This study quantifies the combined health and environmental impacts of foods to guide dietary choices that align with both human and ecological targets.
- Jennings R, Henderson AD, Phelps A, Janda KM, van den Berg AE. Five US dietary patterns and their relationship to land use, water use, and greenhouse gas emissions: Implications for future food security. Nutrients. 2023;15(1):215. 10.3390/nu15010215
  - This cross-sectional study investigates the climate impacts associated with five real-world dietary patterns—healthy US, vegetarian, vegan, Mediterranean, US Dietary Guidelines—and quantifies the contribution of various food groups to each environmental marker.
- Mendoza-Vasconez AS, Landry MJ, Crimarco A, Bladier C, Gardner CD. Sustainable diets for cardiovascular disease prevention and management. Curr Atheroscler Rep. 2021;23(7):31. 10.1007/s11883-021-00929-0
  - Review exploring dietary patterns appropriate for preventing and managing CVD, improving environmental outcomes, and supporting economic sustainability.
- Willett W, Rockström J, Loken B, Springmann M, Lang T, Vermeulen S, Garnett T, Tilman D, DeClerck F, Wood A, Jonell M. Food in the Anthropocene: the EAT–Lancet Commission on healthy diets from sustainable food systems. Lancet. 2019;393(10170):447 − 92. 10.1016/S0140-6736(18)31788-4
  - The EAT-Lancet Commission outlines a healthy reference diet designed to satisfy nutrient requirements and optimize human health while accounting for planetary boundaries and food system practices.

## Supplementary Information

Below is the link to the electronic supplementary material.

Supplementary Material 1 (PDF 205 KB)

## Data Availability

No datasets were generated or analysed during the current study.
