# Supplementary material for: Diets for Dual Cardiovascular and Planetary Health: A Scoping Review
Source: Curr Atheroscler Rep. 2025 Dec 10;27(1):122. doi: 10.1007/s11883-025-01344-5 (PMC12696061; doi:10.1007/s11883-025-01344-5)
Supplement: Supplementary file 1 — Supplementary Material 1 (PDF 205 KB) [file 11883_2025_1344_MOESM1_ESM.pdf]

Supplemental Table 1. Description of Indices

Dietary Pattern of Interest (Aim)	Index/Score Name	Index/Score Description	Components	Total Score (Range)
**EAT-Lancet Diet Recommendations** (Promote human and planetary health)	**Planetary Health Diet (PHD) Score**	Categorizes foods according to three groups: **(1) adequacy, (2) optimum, and (3) moderation**. Consumption of foods in the *adequacy* component is encouraged. In contrast, intake of *moderation* foods should be limited due to their association with the risk of chronic illness. The *optimum* category includes foods where modest intake within a select range can be nutritious [1].<br><br>The analysis conducted by Ye and colleagues standardized energy intake at 2,500 calories per day and did *not* differentiate between whole and refined grains, as the sample's grain intake primarily consisted of refined grains. Rather, *total* grains, such as bread, cereals, rice, and noodles, were considered as a *moderation* component [1].	**Adequacy.**<br>Fruits; Vegetables; Nuts; Legumes; Fish; Unsaturated Fats<br><br>**Optimum.**<br>Potatoes; Eggs; Dairy; Poultry<br><br>**Moderation.**<br>Red Meat; Total Grains; Saturated Fats; Added Sugar	0 - 140
	**Healthy Reference Diet (HRD) Score**<br><br>**EAT-Lancet Diet Index (ELDI) Score**	A proportional scoring method that classifies foods into one of four components: **(1) adequacy, (2) moderation, (3) optimum, and (4) ratio**. The concepts behind the *adequacy*, *moderation*, and *optimum* components are the same as the PHD, but vary in food components.<br><br>In contrast to the PHD, the HRD and ELDI: (1) specify and include whole grains in the *adequacy* component, (2) classify nuts and fish as *optimum* rather than *adequacy* foods, (3) categorize soy foods separately from legumes, and (4) incorporate a *ratio* component [1, 2, 3].<br><br>The ratio component refers to the ratio of unsaturated fats to saturated fats. Added fats included palm oil, unsaturated oils, dairy fats, and lard and tallow. Zero intake of saturated fat or a ratio of at least 13 earns the highest points for the ratio component [2, 3].	**Adequacy.**<br>Fruits; Vegetables; Legumes; Soy Foods; Whole Grains<br><br>**Optimum.**<br>Potatoes; Eggs; Dairy; Chicken/Poultry; Nuts; and Fish<br><br>**Moderation.**<br>Sweeteners; Beef, Lamb, and Pork<br><br>**Unsaturated to Saturated Fats Ratio**.<br>Added Fats	
**Healthy Diet** (Lower risk of chronic disease)	**Alternative Healthy Eating Index 2010 (AHEI-2010)**	Designed to quantify diet quality based on the associations between foods and health-related outcomes. The original AHEI-2010 categorizes foods according to 11 groups, with a maximum score of 110, suggesting perfect dietary adherence. Foods reviewed include: (1) vegetables, (2) fruits, (3) whole grains, (4) nuts and legumes, (5) long chain n-3 fats, (6) polyunsaturated fatty acids (PUFAs), (7) sugary beverages (including fruit juice), (8) red and processed meat, (9) trans fat, (10) sodium, and (11) alcohol, where moderate consumption is recommended [4].<br><br>Musicus et al. removed alcohol-containing beverages from the AHEI-2010 scoring method, resulting in a maximum potential score of 100 [5]. The ten food categories	**Encourage Intake.**<br>Fruits; Vegetables; Whole grains;<br>Nuts and Legumes;<br>Long Chain n-3 Fats; PUFAs	0 - 100[a]

		can further be classified as foods to encourage or limit intake [5, 6].	**Limit Intake.**<br>Red and Processed Meat; Trans Fat; Sodium; Sugary Beverages	
**Plant-Based Diet**<br>(Prioritize foods of plant origin)	**Plant-Based Diet Index (PDI)**	Categorizes foods into one of 18 food groups, which are further categorized as: (1) healthy plant foods, (2) unhealthy plant foods, and (3) animal-based foods. Unlike other indices, the PDI considers tea and coffee intake [7].<br><br>Under the PDI, all plant-based foods (healthy and unhealthy) contribute to a higher score. Consumption of animal foods is penalized, thus lowering the total score.<br><br>Notably, Musicus et al. excluded alcohol-containing beverages and margarine from the PDI, hPDI, and uPDI scoring method [5].	**Healthy Plant Foods**.<br>Fruits; Vegetables; Whole Grains; Nuts; Legumes; Tea & Coffee; Vegetable Oils<br><br>**Unhealthy Plant Foods**.[b]<br>Fruit Juice; Refined Grains; Potatoes; Sugar-Sweetened Beverages; Sweets and Desserts<br><br>**Animal Foods**.<br>Meat (includes Poultry); Fish or Seafood; Eggs; Dairy; Animal Fat; Miscellaneous Animal Foods	18 - 90
	**Healthy Plant-Based Diet Index (hPDI)**	Same food categories as PDI. However, under the hPDI, *only* healthy plant foods increase the score. A higher hPDI score suggests a more nutritious diet and has been associated with a lower incidence of coronary heart disease (CHD) [5, 7].	**Healthy Plant Foods**<br><br>**Unhealthy Plant Foods**<br><br>**Animal Foods**	
	**Unhealthy Plant-Based Diet Index (uPDI)**	Same food categories as PDI. However, under the uPDI, *only* unhealthy plant foods increase the score. A higher uPDI score suggests a less nutritious diet and has been associated with a higher incidence of CHD [5, 7].	**Healthy Plant Foods**<br><br>**Unhealthy Plant Foods**<br><br>**Animal Foods**	
**2015 - 2020 Dietary Guidelines for Americans *plus* Ecological and Economic Wellbeing**<br>(Human, planetary, and economic sustainability)	**Sustainable Diet Index**			0 - 9

Sub-Index: Nutritional Quality	The SDI nutritional quality sub-index was developed by modifying the 2015 Dietary Guideline for Americans Index (2015 DGAI) developed by Jessri et al [8]. The SDI nutritional quality sub-index includes two primary components, comprising (1) the energy-dependent Food Intake sub-score and (2) the Healthy Choice sub-score. In brief, greater consumption of plant-based foods (excluding starchy vegetables, cereals, and added sugar), in addition to fish and seafood, is encouraged. Foods to limit include cereals, dairy, meat, eggs, and starchy vegetables [8, 9, 10].<br><br>Beyond assessing the intake of select food groups (e.g., fruit), the SDI nutritional quality sub-score takes into account the *variety* of fruits and vegetables consumed, dietary fiber density, select nutrient intake (total fat, saturated fat, trans fat, cholesterol intake, and sodium), and alcohol intake, in addition to low-fat dairy and lean meat consumption [9, 10]<br><br>When used in isolation, the nutrition quality sub-index has a total possible score of 21 points, with a higher score implying higher dietary adherence [10]. Within the context of the total SDI score, it contributes between 0 and 3 points [9].	**Encourage Intake (Food Intake sub-score).**<br>Variety of Vegetables and Fruits;<br>Dark Green Vegetables;<br>Red/Orange Vegetables; Legumes;<br>Other Vegetables; Fruits;<br>Fish & Seafood.<br><br>**Recommended "Healthy Choice sub-score" Components**.<br>Whole Grain (% of Cereals);<br>Dietary Fiber Density (grams/1000 kcal);<br>Low-Fat Dairy & Lean Meat Products (%)<br><br>**Components with Select Range.**<br>Total Fat (Percent Energy)<br>*Recommendation:* ≥20%, ≤35%<br><br>**Overconsumption is Discouraged.**<br>Starchy Vegetables; Cereals;<br>Meat and Eggs; Dairy Products;<br>Added Sugar[c]<br><br>**"Healthy Choice sub-score" Components to Limit.**<br>Saturated Fat (Percent Energy);<br>Trans Fat (Percent Energy);<br>Cholesterol Intake (mg/day);<br>Sodium (mg/day);<br>Alcohol (servings/day)
Sub-Index: Environmental Impact Index	Based on life-cycle assessments, this index considers four environmental impacts per kilogram of food, comprising land use (m^2/day), water consumption (Liters/day), energy consumption (MJ/day), and greenhouse gas emissions (GHGe) (kg CO_2 equivalent/day) [9, 11].	The contribution of each food product was inversely related to its environmental impact across the four environmental indicators. The environmental impacts of each specific product were not detailed in the document.
Sub-Index:	Encompasses the annual costs per food frequency questionnaire food item. The	The contribution of each food product

	Dietary Market Price	Ministry of Industry, Tourism and Commerce of Spain was the primary data source. The year of enrollment was considered to assist in controlling for price variations. [9]	was inversely related to its market price. The market price of each specific product was not detailed in the document.	
**Nutrient-Dense Diet** (Optimize nutrient intake and minimize empty calories) [12]	**Nutrient Rich Food 11.3 (NRF11.3) Index[d]**	The Nutrient Rich Food 11.3 (NRF11.3) Index was developed by customizing the 12-component Nutrient Rich Food (NRF) index for a Swedish sample [12, 13]. It incorporates sex-specific dietary recommendations, including those for postmenopausal individuals, as outlined in the Nordic Nutrition Recommendations 2012 and calculates scores according to the methodology developed by Fulgoni and colleagues [14]. The revised index consists of 14 components: (1) protein, (2) fiber, (3) vitamin A (retinol equivalents), (4) vitamin C, (5) vitamin E, (6) calcium, (7) iron, (8) potassium, (9) magnesium, (10) vitamin D, (11) folate, (12) saturated fat, (13) added sugars, and (14) sodium. These components can be further categorized into those that should be consumed in greater quantities and those that should be limited. Notably, among the recommended nutrients, fiber does not have a restricted daily amount [13].	**Components to Encourage.** Protein; Fiber; Vitamin A; Vitamin C; Vitamin E; Calcium; Iron; Potassium; Magnesium; Vitamin D; Folate<br>**Components to Limit.** Saturated Fat; Added Sugars; Sodium	NA

[a] The original AHEI-2010 categorizes foods into 11 groups, with a maximum score of 110. However, the featured score range reflects the method applied by Musicus et al., which excludes alcohol. .

[b] It is essential to recognize that while unhealthy plant-based foods are generally discouraged, the PDI score evaluates adherence to a plant-based diet as a whole, irrespective of the nutrient density of the foods consumed. Thus, unhealthy plant-based options are classified as green within this index.

[c] Under the SDI nutritional quality sub-index, added sugar intake ≥10% of energy intake was given 0 points, contrasting the 2015 DGAI, which applied a cut-off value of ≥9% [8, 10].

[d] Strid and colleagues combined the NRF11.3 scores with dietary greenhouse gas emissions (GHGe) to categorize participants into one of four groups: (1) High nutrient density with high climate impact (HNutr/HClim) diet, (2) High nutrient density with low climate impact (HNutr/LClim) diet, (3) Low nutrient density with high climate impact (LNutr/HClim) diet, and (4) Low nutrient density with low climate impact (LNutr/LClim) diet.

Legend.

Foods or food components that should be:

Green	=	Consumed in greater quantities	Yellow	=	Consumed within a set range	Red	=	Avoided or limited

References

1. Ye YX, Geng TT, Zhou YF, He P, Zhang JJ, Liu G, Willett W, Pan A, Koh WP. Adherence to a planetary health diet, environmental impacts, and mortality in Chinese adults. JAMA Netw Open. 2023;6(10):e2339468-. https://doi.org/10.1001/jamanetworkopen.2023.39468
2. Colizzi C, Harbers MC, Vellinga RE, Verschuren WM, Boer JM, Biesbroek S, Temme EH, van der Schouw YT. Adherence to the EAT-lancet healthy reference diet in relation to risk of cardiovascular events and environmental impact: results from the EPIC-NL cohort. J Am Heart Assoc. 2023;12(8):e026318. https://doi.org/10.1161/JAHA.122.026318
3. Cai H, Talsma EF, Chang Z, Wen X, Fan S, Van't Veer P, Biesbroek S. Health outcomes, environmental impacts, and diet costs of adherence to the EAT-Lancet Diet in China in 1997–2015: a health and nutrition survey. Lancet Planet Health. 2024;8(12):e1030-42. https://doi.org/10.1016/S2542-5196(24)00285-7
4. Chiuve SE, Fung TT, Rimm EB, Hu FB, McCullough ML, Wang M, Stampfer MJ, Willett WC. Alternative dietary indices both strongly predict risk of chronic disease. The J Nutr. 2012; 142(6):1009–1018. https://doi.org/10.3945/jn.111.157222
5. Musicus AA, Wang DD, Janiszewski M, Eshel G, Blondin SA, Willett W, Stampfer MJ. Health and environmental impacts of plant-rich dietary patterns: a US prospective cohort study. Lancet Planet Health. 2022;6(11):e892-900. https://doi.org/10.1016/S2542-5196(22)00243-1
6. Al-Ibrahim AA, Jackson RT. Healthy eating index versus alternate healthy index in relation to diabetes status and health markers in U.S. adults: NHANES 2007-2010. Nutr J. 2019;18(1),26. https://doi.org/10.1186/s12937-019-0450-6
7. Satija A, Bhupathiraju SN, Spiegelman D, Chiuve SE, Manson JE, Willett W, Rexrode KM, Rimm EB, Hu FB. Healthful and Unhealthful Plant-Based Diets and the Risk of Coronary Heart Disease in U.S. Adults. *J Am Coll Cardiol*. 2017;*70*(4):411–422. https://doi.org/10.1016/j.jacc.2017.05.047
8. Jessri M, Lou WY, L'Abbé MR. The 2015 Dietary Guidelines for Americans is associated with a more nutrient-dense diet and a lower risk of obesity. Am J Clin Nutr. 2016;104(5):1378-1392. https://doi.org/10.3945/ajcn.116.132647.
9. Fresán U, Martínez-González MA, Segovia-Siapco G, Sabaté J, Bes-Rastrollo M. A three-dimensional dietary index (nutritional quality, environment and price) and reduced mortality: The "Seguimiento Universidad de Navarra" cohort. Prev Med. 2020;137:106124. https://doi.org/10.1016/j.ypmed.2020.106124.
10. Fresán U, Sabaté J, Martínez-Gonzalez MA, Segovia-Siapco G, de la Fuente-Arrillaga C, Bes-Rastrollo M. Adherence to the 2015 Dietary Guidelines for Americans and mortality risk in a Mediterranean cohort: The SUN project. Prev Med. 2019;118:317-324. https://doi.org/10.1016/j.ypmed.2018.11.015
11. Fresán U, Martínez-Gonzalez MA, Sabaté J, Bes-Rastrollo M. The Mediterranean diet, an environmentally friendly option: evidence from the Seguimiento Universidad de Navarra (SUN) cohort. Public Health Nutr. 2018;21(8):1573-1582. https://doi.org/10.1017/S1368980017003986.
12. Drewnowski A. Defining nutrient density: development and validation of the nutrient rich foods index. J Am Coll Nutr. 2009;28(4):421S-426S. https://doi.org/10.1080/07315724.2009.10718106

13. Strid A, Johansson I, Lindahl B, Hallström E, Winkvist A. Toward a more climate-sustainable diet: possible deleterious impacts on health when diet quality is ignored. J Nutr. 2023;153(1):242-52. https://doi.org/10.1016/j.tjnut.2022.10.004
14. Fulgoni VL 3rd, Keast DR, Drewnowski A. Development and validation of the nutrient-rich foods index: a tool to measure nutritional quality of foods. J Nutr. 2009;139(8):1549-54. https://doi.org/10.3945/jn.108.101360
